# Biocompatibility of Nickel Ferrite Nanoparticles on Systemic and Testicular Cells

**DOI:** 10.1021/acsomega.5c09491

**Published:** 2026-01-06

**Authors:** Carla Cristina Martins Silva, Pedro Igor Macário Viana, Thalita Marcolan Valverde, José Domingos Ardisson, Daniele Alves Fagundes, Guilherme Mattos Jardim Costa

**Affiliations:** † Laboratório de Biologia Celular, Instituto de Ciências Biológicas, 28114 Universidade Federal de Minas Gerais—UFMG, Belo Horizonte, Minas Gerais 31270-901, Brasil; ‡ Laboratório de Síntese de Nanoestruturas, 54530 Centro de Desenvolvimento da Tecnologia Nuclear, Belo Horizonte, Minas Gerais 31270-901, Brasil

## Abstract

Nickel ferrite nanoparticles (NPs) are chemically stable and have a surface suitable for functionalization, making them potential biotechnological tools in male reproduction. Herein, the nickel ferrite (FeNi) NPs were synthesized by the hydrothermal method and presented colloidal stability, low aggregation, high crystallinity, and superparamagnetic behavior at room temperature. At a dose of 500 μg/mL, FeNi NPs reduced the viability of systemic and male reproductive cells at all time points. We selected 100 μg/mL to further investigate its effects on testicular cells, as it was safe for VERO and AML-12 cells. For male reproductive cells, we observed that the selected FeNi NP dose significantly increased cell death by apoptosis and necrosis in Leydig cells, and by apoptosis in germ cells. We observed rapid internalization of FeNi NPs in both cell types within the first 5 min of exposure. Transmission electron microscopy confirmed their presence in the cytoplasm and within vesicles, suggesting internalization via passive diffusion and endocytosis. Additionally, phagosome formation was noted in TM3 cells. The rapid and extensive internalization of these nanoparticles within testicular cells may result in cellular apoptosis. We also observed an increase in reactive oxygen species after exposure of male reproductive cells to FeNi NP. These findings provide a foundation for future investigations into the biomedical applications of FeNi nanoparticles in reproductive cells.

## Graphical abstract

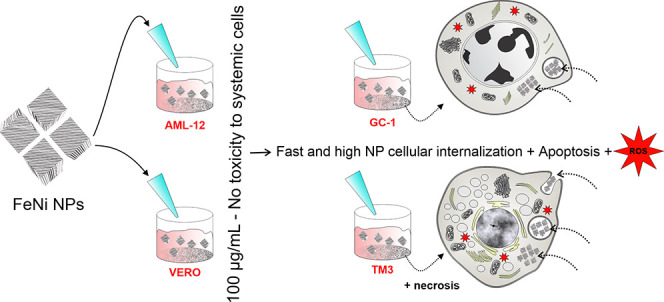

## 1 Introduction

In recent years, the application of nanomaterials with dimensions ranging from 1 to 100 nm^1–3^ in various biomedical fields has grown exponentially, contributing to significant advancements in health.^4,5^ However, this progress has raised concerns about the potential toxic effects of these materials, including DNA damage, oxidative stress, and inflammation.^3,6^

Although the effects of nanoparticles (NPs) on organs such as the liver and kidneys are well-documented in the literature, including bioaccumulation, increased reactive oxygen species (ROS), functional and histological alterations, increased nitric oxide, lipid peroxidation, suppression of antioxidant enzymes, apoptosis, and hormonal dysregulation,^7–9^ understanding the impact of NPs on reproductive organs remains a relatively recent area of research.^10^

Among the various classes of inorganic nanoparticles, metal-containing nanoparticles such as those made from iron,^11^ silver,^12^ gold,^13^ and nickel^14–16^ stand out. These nanoparticles are being explored for their unique physicochemical properties and their applications in areas like controlled drug delivery and targeted therapies. Specifically, ferrite nanoparticles have garnered interest due to their magnetic properties, chemical stability, and relevance in diverse technical applications, such as magnetic hyperthermia.^6,17–19^

These inorganic nanoparticles can be synthesized using various methods, such as coprecipitation,^20^ sol–gel,^21^ microemulsion,^22^ thermal decomposition,^23^ and hydrothermal methods.^24^ The choice of method directly influences characteristics such as size, morphology, purity, and the structural order of the nanomaterial.^25^ Among these approaches, hydrothermal synthesis offers distinct advantages for biomedical applications: it produces nanoparticles with high purity and crystallinity, ensuring superior chemical stability, functional performance, and reduced toxicity.^18,19,26^ Unlike thermal decomposition or microemulsion methods, hydrothermal synthesis eliminates the need for organic solvents, thereby avoiding surface contamination and toxic residues.^27^ Compared to coprecipitation, it yields nanoparticles with narrower size distribution, and improved structural homogeneity.^28^

Among nanomaterials with biomedical potential, nickel ferrites (NiFe_2_O_4_) stand out.^19^ These nanoparticles exhibit superparamagnetic properties, chemical stability, superior magnetic properties, and a surface compatible with functionalization.^29^ Although nickel exhibits significant toxicity nanostructuring nickel into ferrite nanoparticles can reduce systemic toxicity, making it a promising candidate for biomedical applications.^30^ However, studies evaluating the biological impact of nickel ferrite in nanoparticle form remain scarce, highlighting the need to investigate its biocompatibility in cell cultures, as well as the mechanisms of toxicity and interactions with cellular structures. This interest is particularly relevant because studies have already shown that other inorganic nanoparticles, such as iron oxide nanoparticles, can alter male reproductive function.^31^

Considering this context, the main objective of this study was to synthesize, characterize, and evaluate the toxicological effects of nickel ferrite nanoparticles on immortalized cell lines derived from the liver, kidney, and male reproductive system. This evaluation facilitated a comprehensive study of the biocompatibility and potential toxicity mechanisms associated with these nanomaterials, thereby ensuring the safe development for biomedical applications.

## 2 Materials and Methods

### 2.1 Nanoparticle Synthesis

Nickel ferrite (FeNi) nanoparticles (NPs) were synthesized using the hydrothermal method with iron­(III) nitrate nonahydrate—Fe­(NO_3_)_3_·9H_2_O, nickel­(II) nitrate hexahydrate—Ni­(NO_3_)_2_·6H_2_O, sodium hydroxide pellets—NaOH, and atmospheric oxygen—O_2_. The quantity of reagents used was determined based on the reaction stoichiometry, and the synthesis was conducted to obtain 1 g of the nanomaterial. The procedure was carried out in an autoclave at 130 °C and 2 bar of pressure, with a reaction time of 2 h. The resulting precipitate was washed with Milli-Q water until reaching a pH of 7 and subsequently dried in an oven at 100 °C for 4 h.^19^ All reagents used were of analytical grade, acquired from Sigma-Aldrich (St. Louis, MO, USA), and employed without any additional purification.

### 2.2 Nanoparticle Characterization

After synthesizing the nanomaterial, it was characterized to obtain details about its structural and magnetic properties. X-ray diffraction (XRD) was conducted to confirm the crystalline structure of the nanoparticles using the powder method with copper Kα radiation (λ = 1.54178 Å). The analysis was performed at an Ultima IV diffractometer system (Rigaku Corporation, Tokyo, Japan) with a scanning speed of 1° (2θ)/min over the range of 15° to 70°. Transmission electron microscopy (TEM) was employed to evaluate the morphology of the NP using a TECNAI G2-20—Supertwin FEI transmission electron microscope (Hillsboro, Oregon, USA) operating at 200 kV. Based on the TEM images, a histogram of particle size distribution was generated, considering the average diameter of 500 particles, using the Quantikov.5 software.

Fourier-transform infrared spectroscopy (FTIR) was conducted to investigate chemical bonds using the Nicolet 6700 spectrometer (Thermo Fisher Scientific, Waltham, MA, USA) with the attenuated total reflectance (ATR) method. A total of 64 scans were performed in the range of 2000–400 cm^–1^, with a resolution of 4 cm^–1^. The chemical composition of the FeNi NP was investigated through X-ray fluorescence (XRF) using the Geigerflex spectrometer (Rigaku Corporation, Tokyo, Japan), allowing for qualitative and quantitative identification of the elements present in the sample. To analyze the surface chemical composition of the NP, X-ray photoelectron spectroscopy (XPS) was performed using the SPECS system (Berlin, Germany) equipped with a Phoibos-150 analyzer and a monochromatic X-ray source of Al Kα radiation (1486.6 eV).

The magnetic properties of the nanoparticles were evaluated using a vibrating sample magnetometer (VSM) in a LakeShore 7404 magnetometer (Westerville, OH, USA). Mössbauer spectra were collected in transmission mode using a ^57^Co source in a Rh matrix at room temperature and at low temperature (40 K) to minimize thermal noise and ensure accurate determination of hyperfine parameters. To measure the hydrodynamic size and zeta potential of the nanoparticles, we used two methods: dynamic light scattering (DLS) and electrophoretic light scattering. Initially, the nanoparticles were dispersed in sterile injection-grade water and subjected to ultrasonic exposure for 70 min. DLS was also performed with nanoparticles dispersed in cell culture medium supplemented with fetal bovine or equine serum, depending on the cell type. The concentration used for measurements was 100 μg/mL, and the Zetasizer Nano ZS 3000 HSA (Malvern Panalytical, Malvern, Worcestershire, UK) was used for the analyses. Each test was conducted in triplicate, and the results were expressed as mean ± standard deviation.

### 2.3 Cell Culture

For biological assays, the following immortalized cell lines were used: hepatic cells (AML-12, ATCC CRL-2254), originating from hepatocytes isolated from the liver of a 3 month-old normal mouse; renal cells (VERO, ATCC CCL-81), derived from renal epithelial cells of an African green monkey; Leydig cells (TM3, ATCC CRL-1714), isolated from a male mouse; and germ cells (GC-1, ATCC CRL-2053), isolated from the testis of a 10 day-old male mouse.

The AML-12 cells were cultured in Dulbecco’s Modified Eagle Medium/Nutrient Mixture F-12 (DMEM/F-12) supplemented with 10% fetal bovine serum (FBS), 1% antibiotic (streptomycin −100 μg/mL, penicillin −500 U/mL), 1% insulin transferrin selenium (ITS), and 20 ng/mL dexamethasone. The VERO cells were cultured in Alpha Minimum Essential Medium (α-MEM) supplemented with 10% FBS and 1% antibiotic. The TM3 cells were also cultured in DMEM/F-12, but supplemented with 5% horse serum (HS), 2.5% FBS, and 1% antibiotics. Finally, GC-1 cells were cultured in Dulbecco’s Modified Eagle Medium (DMEM) supplemented with 10% FBS and 1% antibiotics. The FBS, horse serum, and α-MEM were obtained from Nova Biotecnologia (Cotia, São Paulo, Brazil). The antibiotics (streptomycin and penicillin) were acquired from Thermo Fisher Scientific (Waltham, MA, USA), while DMEM and DMEM/F-12 media were purchased from Gibco (Grand Island, NY, USA). All cells were maintained in a humidified incubator with a 5% CO_2_ atmosphere at 37 °C. Subculturing was performed every 2 days, following the recommended splitting ratios provided by the American Type Culture Collection (ATCC).

### 2.4 Sample Preparation

For biological assays, the NPs were weighed on a precision balance and prepared as a stock suspension at a concentration of 5000 μg/mL. For this, 10 mg of nickel ferrite was added to 2 mL of sterile injection-grade water. The suspension was dispersed in an ultrasonic bath without heating for 70 min and subsequently added, at the desired proportions, to a cell culture medium for in vitro testing.

### 2.5 Cell Viability

The CellTiter-Blue viability assay (Promega, Madison, USA) was used to assess how different cell lines respond to nickel ferrite nanoparticles. Initially, after thawing and expansion, AML-12, VERO, TM3, and GC-1 cells were seeded into 96-well plates at a density of 1 × 10^4^ cells/well in biological sextuplicates for 24 h. In order to evaluate the toxicity of nickel ferrite and to determine the appropriate dosage for subsequent experiments, we conducted an analysis of seven concentrations: 6.25, 12.5, 25, 50, 100, 250, and 500 μg/mL.^14^ We analyzed the cells treated with these concentrations at three time points: 24, 48, and 72 h.

To ensure the reliability of the assay, both viability (negative) and cytotoxicity (positive) controls were included. The negative control was treated with a culture medium containing sterile injection-grade water, which was used to disperse the nanoparticles. For the positive cell death control, cells were treated with 0.1% Triton X-100 (Sigma-Aldrich, St. Louis, USA) for 5 min after 24, 48, and 72 h of treatment. A total of 20 μL of medium was removed from each well, and 20 μL of CellTiter-Blue reagent was added. The plates were incubated for 4 h in a humidified atmosphere with 5% CO_2_ at 37 °C. Fluorescence readings were performed using the Cytation 5 Cell Imaging Reader - BioTek, with excitation at 560/20 nm and emission at 590/20 nm.

Cell viability was assessed in biological sextuplicates, based on the conversion of resazurin to resorufin by metabolically active cells, with results normalized to the viability control group and expressed as percentages using GraphPad Prism 8.4 software.^32^ Half-maximal inhibitory concentration (IC_50_) values were determined to assess dose-dependent cytotoxicity. Cell viability data were transformed logarithmically and fitted to sigmoidal dose–response curves using nonlinear regression. The IC_50_, representing the concentration that reduces cell viability by 50%, was calculated for each cell line at 24, 48, and 72 h of exposure.

### 2.6 Cell Death

To investigate the pathway of cell death, TM3 and GC-1 cells were seeded into 6-well plates at a density of 2 × 10^5^ cells/well, in biological triplicates, and incubated in a 5% CO_2_ atmosphere at 37 °C. After 24 h, cells were treated with FeNi NPs at a concentration of 100 μg/mL, while the negative control received only sterile injection-grade water. After 72 h of treatment, the cells were resuspended and stained using the Alexa Fluor 488 Annexin V/Dead Cell Apoptosis Kit (Thermo Fisher Scientific) according to the manufacturer’s instructions. The samples were then analyzed using the FACSCalibur flow cytometer equipped with a 15 mW argon laser (excitation at 488 nm), acquiring 10,000 events per sample, and data were processed using FlowJo software (Version 10.10.0).^32–34^ After analysis, images of Annexin V/PI-stained cells were obtained at 10× magnification using the Cytation 5 Cell Imaging Reader - BioTek. The images presented provide a qualitative representation of necrotic cells identified by propidium iodide (PI) staining and apoptotic cells marked by Annexin V labeling.

### 2.7 Internalization Kinetics

The internalization kinetics of NPs in cells were evaluated using the Magnetic-Activated Cell Sorting (MACS) system with the OctoMACS Kit (Miltenyi Biotec). TM3 and GC-1 cells were seeded into 12-well plates at a density of 2 × 10^4^ cells/well, in biological triplicates, and incubated in a humidified 5% CO_2_ atmosphere at 37 °C. After 24 h, the culture medium was removed from each well and replaced with fresh medium containing FeNi NPs at a concentration of 100 μg/mL. The plates were then incubated for 5, 10, 20, 40, 60, 120, and 180 min to allow internalization of the nanoparticles by the cells. Internalization was also evaluated at 24, 48, and 72 h to determine whether nanoparticle uptake patterns remained stable throughout the experimental period used for cytotoxicity assays.

At each time point, the cells were detached using 0.25% trypsin with 1 mM EDTA (Gibco, Grand Island, NY, USA). The cell suspension was homogenized, resuspended, and added to the magnetic column in conjunction with the OctoMACS separator. Cells that internalized magnetic nanoparticles were retained within the column, whereas noninternalized cells were able to pass through freely. We counted the cells using a Neubauer chamber and found the average number of cells that internalized the nanoparticles and those that did not for each well and exposure time. The results were expressed as percentages.

### 2.8 Transmission Electron Microscopy

To detect intracellular nanoparticles, TM3 and GC-1 cells were cultured in T75 flasks. Following subculture, the cells were maintained in flasks for 48 h, reaching approximately 90% confluence, as recommended by ATCC. The medium in the flasks was then removed and replaced with culture medium containing sterile injection-grade water for the control group, and culture medium containing FeNi NPs at a concentration of 100 μg/mL for the ferrite group. The flasks were incubated in a 5% CO_2_ incubator at 37 °C for 3 h. After the incubation time, the medium was removed, and the monolayer was washed with basal medium to remove dead cells and sedimented nanoparticles. The cells were then fixed with 2.5% glutaraldehyde (Merck, Darmstadt, Germany) in phosphate buffer (Merck, Darmstadt, Germany) and stored in the refrigerator overnight.

The following day, the fixative was carefully removed from the flask, and the cells were subsequently detached utilizing a cell scraper (KASVI, Pinhais, Brazil). The cells were transferred to Eppendorf tubes containing phosphate buffer and centrifuged at 1400 rpm for 7 min. The resulting pellet was sent to the Microscopy Center at UFMG for preparation for transmission electron microscopy (TEM) analysis, following the steps of resin embedding, ultrathin sectioning, and contrast staining. The analysis was conducted utilizing a TECNAI G2–20 Supertwin FEI transmission electron microscope (TEM) operating at a voltage of 80 kV. To validate the presence of nanoparticles within the cells, energy dispersive spectroscopy (EDS) was employed, allowing for the identification of the chemical elements present in the observed field.

### 2.9 Reactive Oxygen Species (ROS) Production

Reactive oxygen species (ROS) production was evaluated using the ROS Detection Assay Kit (DCFDA/H_2_DCFDA, Invitrogen). TM3 and GC-1 cells were seeded in 96-well plates at a density of 1 × 10^4^ cells/well in biological sextuplicates. Twenty-4 h after plating, control groups received culture medium with injection-grade water (negative control) or hydrogen peroxide (H_2_O_2_, 100 μM; positive control), while the treatment group received culture medium containing FeNi nanoparticles (100 μg/mL).

After 72 h of exposure, cells were labeled with CM-H_2_DCFDA according to the manufacturer’s instructions. Fluorescence was measured using a Cytation 5 Cell Imaging Reader (BioTek) at 495 nm (excitation) and 520 nm (emission). Quantitative analysis was performed on the acquired images, and qualitative assessment of ROS production was conducted by visualizing green fluorescent signals at 20× magnification. Data were expressed as relative fluorescence units (RFU) normalized to the negative control (adapted from Dantas et al. 2024).^35^

### 2.10 Statistical Analyses

The data were assessed for normality using the Shapiro–Wilk test. Results are presented as mean ± standard deviation (SD). Statistical analyses were conducted using GraphPad Prism version 8.4 (GraphPad Software Inc., San Diego, CA, USA). For comparisons involving only two groups, specifically the control group and the FeNi NP-treated group, the student’s *t*-test was employed. In instances where the analysis included more than two experimental groups, a one-way analysis of variance (ANOVA) was conducted for parametric data, followed by Dunnett’s post hoc test. Conversely, for nonparametric data, the Kruskal–Wallis test was utilized, succeeded by Dunn’s post hoc test for further analysis. Differences were considered statistically significant when *p* ≤ 0.05.

## 3 Results

### 3.1 Characterization of Nickel Ferrite Nanoparticles

Following the synthesis of the nanomaterial, nickel ferrite nanoparticles were successfully obtained, and their crystalline structure was confirmed through X-ray diffraction (XRD) analysis (Figure 1A). The observed diffraction peaks were characteristic of cubic ferrite structures, as specified by the reference sheet No. 00-054-0964 for nickel ferrite provided by the International Centre for Diffraction Data, demonstrating the material’s crystallinity. The crystallite size was estimated using Scherrer’s equation^36^ to be 13 ± 1 nm, and the lattice parameter was measured to be 8.35 ± 0.01 Å, which is close to the theoretical value of 8.33 Å for nickel ferrite.^37^ Transmission electron microscopy (TEM) analysis (Figure 1B) revealed that the nanoparticles exhibited a morphology ranging from spherical to cubic, with an average particle size of 13.0 ± 4.7 nm and a unimodal size distribution (Figure 1C).

**Figure 1:**
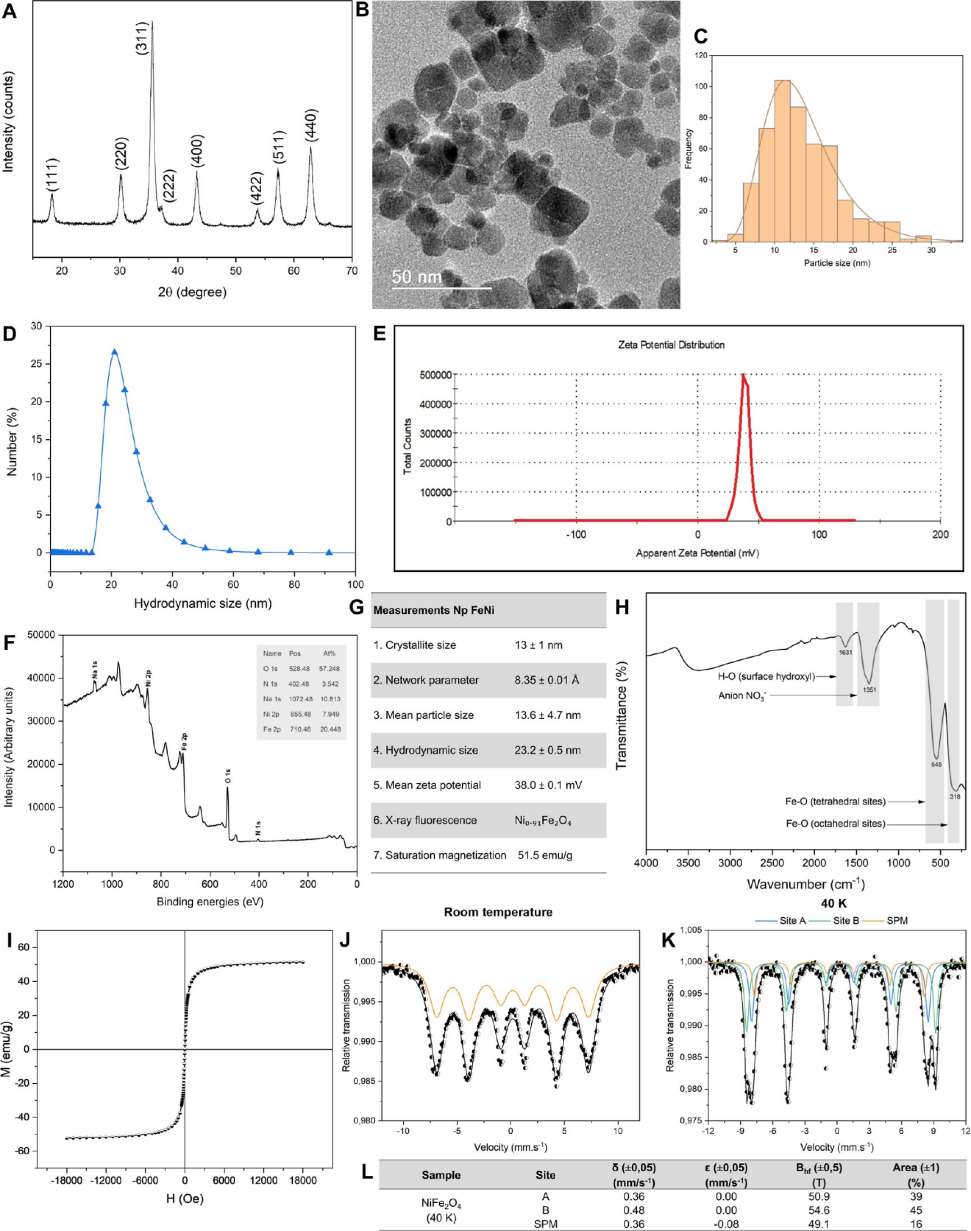
Characterization of nickel ferrite nanoparticles. (A) X-ray diffraction showing the characteristic peaks of the cubic structure of nickel ferrite. (B) Transmission electron microscopy (TEM) image, demonstrating that the morphology of the nanoparticles varies from spherical to cubic. (C) Histogram of particle size distribution, with an average size of 13 ± 1 nm. (D) Hydrodynamic particle size in solution, with an average of 23.2 ± 0.5 nm, demonstrating a low degree of aggregation. (E) Zeta potential with an average of 38.0 ± 0.1 mV, indicating solution stability. (F) X-ray photoelectron spectroscopy (XPS) showing the presence of surface sodium. (G) Representation of FeNi nanoparticle measurements: Crystallite size (G.1), Lattice parameter (G.2), Average particle size (G.3), Hydrodynamic size (G.4), Zeta potential (G.5), X-ray fluorescence (G.6) and saturation magnetization (G.7). (H) Fourier-transform infrared spectroscopy (FTIR) in the infrared region. (I) Hysteresis curve confirming superparamagnetic behavior. (J) Mössbauer spectrum obtained at room temperature, with line broadening of hyperfine fields due to superparamagnetic behavior. (K) Mössbauer spectrum at 40 K, resolving the tetrahedral (A) and octahedral (B) sites, in addition to evidencing particles that remain superparamagnetic (SPM). (L) Mössbauer spectrum measurements performed at 40 K, confirming the expected parameters for the cubic structure of nickel ferrite. The results are presented as mean ± standard deviation.

In contrast, the mean hydrodynamic size of the nanoparticles in injection water was measured at 23.2 ± 0.5 nm (Figure 1D), indicating a low degree of aggregation in the dispersions prepared for biological assays. The zeta potential averaged 38.0 ± 0.1 mV (Figure 1E), reflecting the stability of the solution, which may have been enhanced by the surface presence of sodium, as confirmed by X-ray photoelectron spectroscopy (XPS) (Figure 1F). In cell culture medium, a slight increase in hydrodynamic size was observed compared to measurements in injection-grade water, but values remained below 50 nm (Supplementary Figure 1A,B), indicating minimal aggregation. The zeta potential shifted to −7.8 to −11.3 mV (Supplementary Figure 1B), reflecting reduced colloidal stability, likely due to interactions between nanoparticles and serum proteins in the culture medium.^38,39^

A summary of the mentioned FeNi NPs measurements is represented in the graphs shown in Figure 1G. X-ray fluorescence analysis indicated that the synthesized NPs exhibit a stoichiometry corresponding to Ni_0_._91_Fe_2_O_4_ (Figure 1G). Given that no secondary phases were detected in the structural analyses. The slight stoichiometric deviation is likely associated with differences in precipitation kinetics and ionic mobility between Fe^3+^ and Ni^2+^ cations under the employed hydrothermal conditions. In the infrared spectrum (Figure 1H), the band at 1631 cm^–1^ can be attributed to the stretching and bending modes of the H–O bond associated with probable surface hydroxyl groups and adsorbed water molecules.^40,41^ The band at 1351 cm^–1^ is associated with stretching vibrations of N–O bonds,^42^ possibly resulting from nitrate precursors used during the synthesis process. The bands observed at 545 cm^–1^ and 318 cm^–1^ correspond to metal–oxygen bond vibrations in the tetrahedral and octahedral sites of the cubic ferrite structure, respectively.^43^

### 3.2 Superparamagnetic Characteristic of FeNi NPs

According to the hysteresis curve presented in Figure 1I, both the coercive field and the remanent magnetization were observed to be nearly zero. This finding indicates the superparamagnetic behavior of the FeNi nanoparticles at room temperature. The saturation magnetization value was 51.5 emu/g (Figure 1G), which is close to the bulk material value (∼50 to 55 emu/g) reported by Nejati and Zabihi (2012),^18^ indicating the crystallinity of the synthesized material.

Mössbauer spectra were obtained at room temperature (Figure 1J) and at 40 K (Figure 1K). At room temperature, the observed broadening of spectral lines suggests a partial collapse of hyperfine fields within the tetrahedral (A) and octahedral (B) sites of the ferrites. This phenomenon is likely due to the diminished size of the nanoparticles and their superparamagnetic behavior at this temperature.^44,45^ In order to mitigate the superparamagnetic effect and clarify the hyperfine magnetic structure of FeNi nanoparticles, measurements were conducted at 40 K. The low-temperature spectrum was analyzed and fitted with two sextets, which correspond to the presence of Fe^3+^ ions in both tetrahedral (A) and octahedral (B) sites. Additionally, a third broad sextet was identified, indicative of very small particles exhibiting superparamagnetic behavior even at 40 K. The hyperfine parameter values (see Figure 1L) align with those documented in the literature for nickel ferrite,^46–48^ thereby confirming the cubic structure identified in the X-ray diffraction (XRD) analysis.

### 3.3 Cytotoxicity of FeNi NPs in Systemic and Male Reproductive Cells

After exposure to FeNi NPs for 24, 48, and 72 h, it was observed that VERO cells exhibited viability below 70% (red line) at concentrations of 250 μg/mL and 500 μg/mL (Figure 2A) across all three time points. The administration of 100 μg/mL showed significant differences compared to the control at all three time points, while 50 μg/mL exhibited significant differences at 48 and 72 h. Both doses maintained viability above 70% (Figure 2A).

**Figure 2:**
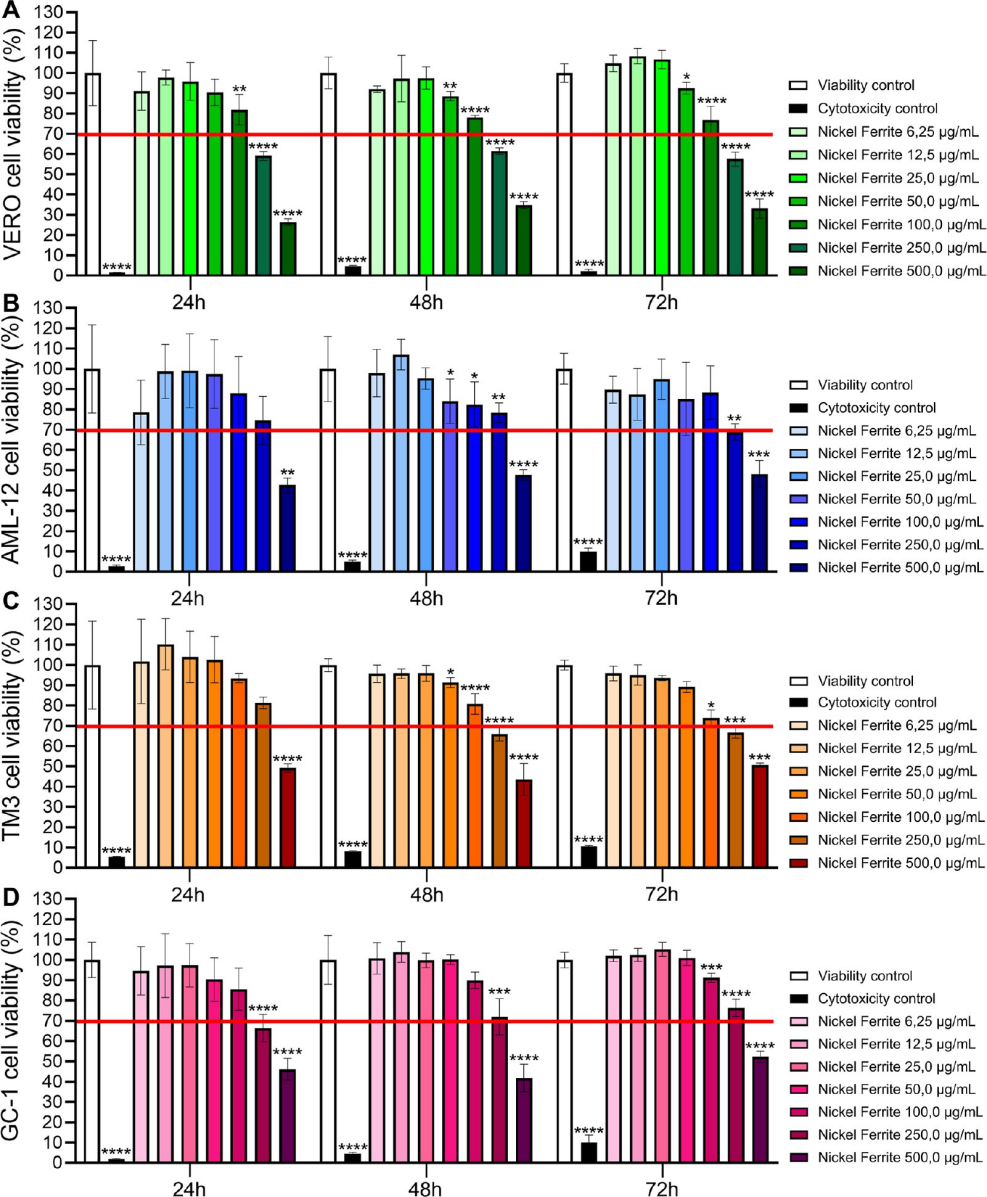
Viability of kidney, liver, Leydig, and germ cells exposed to nickel ferrite nanoparticles. Viability of VERO cells (A), AML-12 cells (B), TM3 cells (C), and GC-1 cells (D) exposed to FeNi NPs at different concentrations (6.25, 12.5, 25, 50, 100, 250, and 500 μg/mL) and at different time points (24, 48, and 72 h). Statistical analyses used one-way ANOVA with Dunnett’s test for parametric data and Kruskal–Wallis with Dunn’s post hoc test for nonparametric data. Results are presented as mean ± SD (*n* = 6). Significant differences are noted as (*) *p* ≤ 0.05, (**) *p* ≤ 0.01, (***) *p* ≤ 0.001, (****) *p* < 0.0001. The red line indicates the 70% viability threshold per ISO 10993–5.

In contrast, AML-12 cells displayed significant differences at a dose of 250 μg/mL at 48 and 72 h, with cytotoxicity observed at 250 μg/mL only at 72 h and at 500 μg/mL across all time points (Figure 2B). Likewise, the doses of 50 μg/mL and 100 μg/mL showed significant differences compared to the control at 48 h, with both maintaining over 70% viability (Figure 2B).

In the investigation of TM3 cells, notable differences in cell viability were identified only at a concentration of 500 μg/mL after 24 h of exposure (Figure 2C). After 48 h, we noticed changes at doses over 50 μg/mL. After 72 h, differences appeared at doses above 100 μg/mL. The administration of 500 μg/mL was determined to be toxic at all three time points (24, 48, and 72 h), while a dosage of 250 μg/mL was deemed toxic after 48 and 72 h (Figure 2C).

In addition, with respect to GC-1 cells, significant statistical differences were noted at concentrations of 250 μg/mL and 500 μg/mL after 24 and 48 h of exposure. Furthermore, at the 72 h mark, significant differences were identified at doses of 100 μg/mL, 250 μg/mL, and 500 μg/mL. Cell viability was notably impacted at 250 μg/mL after 24 h, and at 500 μg/mL across all three time intervals (Figure 2D).

We conducted a viability analysis and set a target dose based on a 70% viability threshold to ensure nontoxicity, following ISO 10993-5, 2009 guidelines.^49^ We selected a dose of 100 μg/mL to minimize harmful effects on systemic cells and proceeded with further studies using TM3 and GC-1 cells. The IC_50_ values were determined for all cell lines at 24, 48, and 72 h of exposure, as detailed in Supplementary Table 1. VERO cells exhibited the lowest IC_50_ values, ranging from 283.4 to 323.4 μg/mL, indicating a higher sensitivity to FeNi nanoparticles. Conversely, reproductive cells (TM3 and GC-1) and hepatocytes (AML-12) demonstrated elevated IC_50_ values, between 418.4 and 549.0 μg/mL, reflecting reduced cytotoxic sensitivity. Overall, IC_50_ values remained relatively stable across the time points for each cell type, with no significant temporal trends identified.

### 3.4 FeNi Nanoparticles Induce Cell Death in Reproductive Cells

A substantial decrease in cell viability was observed following 72 h of exposure to FeNi nanoparticles. Consequently, this time point was selected for the assessment of the cell death pathway. In TM3 cells, there was a significant difference in the number of viable cells in the ferrite-treated group compared to the control group (Figure 3A). Additionally, the ferrite-treated group showed an increase in apoptosis, necrosis, and double labeling, with both necrosis and apoptosis, compared to the negative control (Figure 3B). In GC-1 cells, a significant difference in cell viability was observed in the treated group compared to the control (Figure 3C), along with an increase in apoptotic cells (Figure 3D).

**Figure 3:**
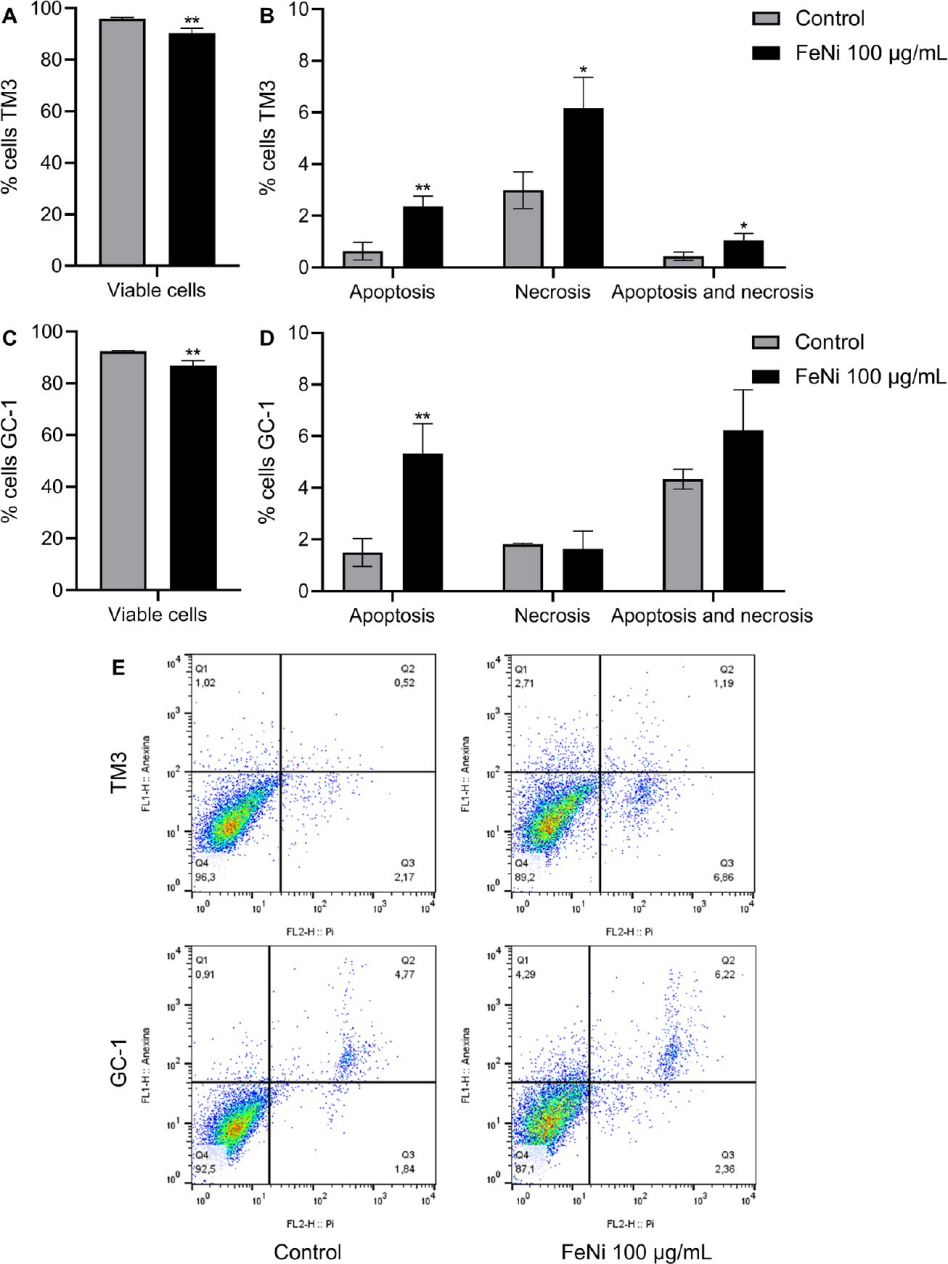
Cell death by apoptosis in GC-1 and TM3 cells, and necrosis in TM3 cells after 72 h of nickel ferrite nanoparticle exposure. (A) Reduction in cell viability in TM3 cells. (B) Percentage of apoptotic and necrotic cells in TM3, identified by Annexin V and propidium iodide (PI) staining. (C) Reduction in cell viability in GC-1 cells. (D) Percentage of apoptotic and necrotic cells in GC-1, identified by Annexin V and PI staining. (E) Dot plots of cell populations analyzed by flow cytometry for TM3 and GC-1 cells, showing viable cells (Q4 quadrant), necrotic cells (Q3 quadrant), apoptotic cells (Q1 quadrant), and cells with both apoptotic and necrotic markers (Q2 quadrant). Results are presented as mean ± SD (*n* = 3). Statistical analysis was performed using the *t*-test, with significant differences considered at *p* ≤ 0.05, represented as follows: (*) *p* ≤ 0.05 and (**) *p* ≤ 0.01.

In the dot plots generated by flow cytometry, viable cells are identified in the Q4 quadrant, necrotic cells are located in the Q3 quadrant, apoptotic cells are represented in the Q1 quadrant, and cells exhibiting dual labeling (apoptosis and necrosis) are found in the Q2 quadrant (Figure 3E). In these analyses, apoptotic cells marked with Annexin V are discernible through green fluorescence, whereas necrotic cells, labeled with propidium iodide (PI), appear in red. The described results are related to TM3 cells (Figure 4A) and GC-1 cells (Figure 4B).

**Figure 4:**
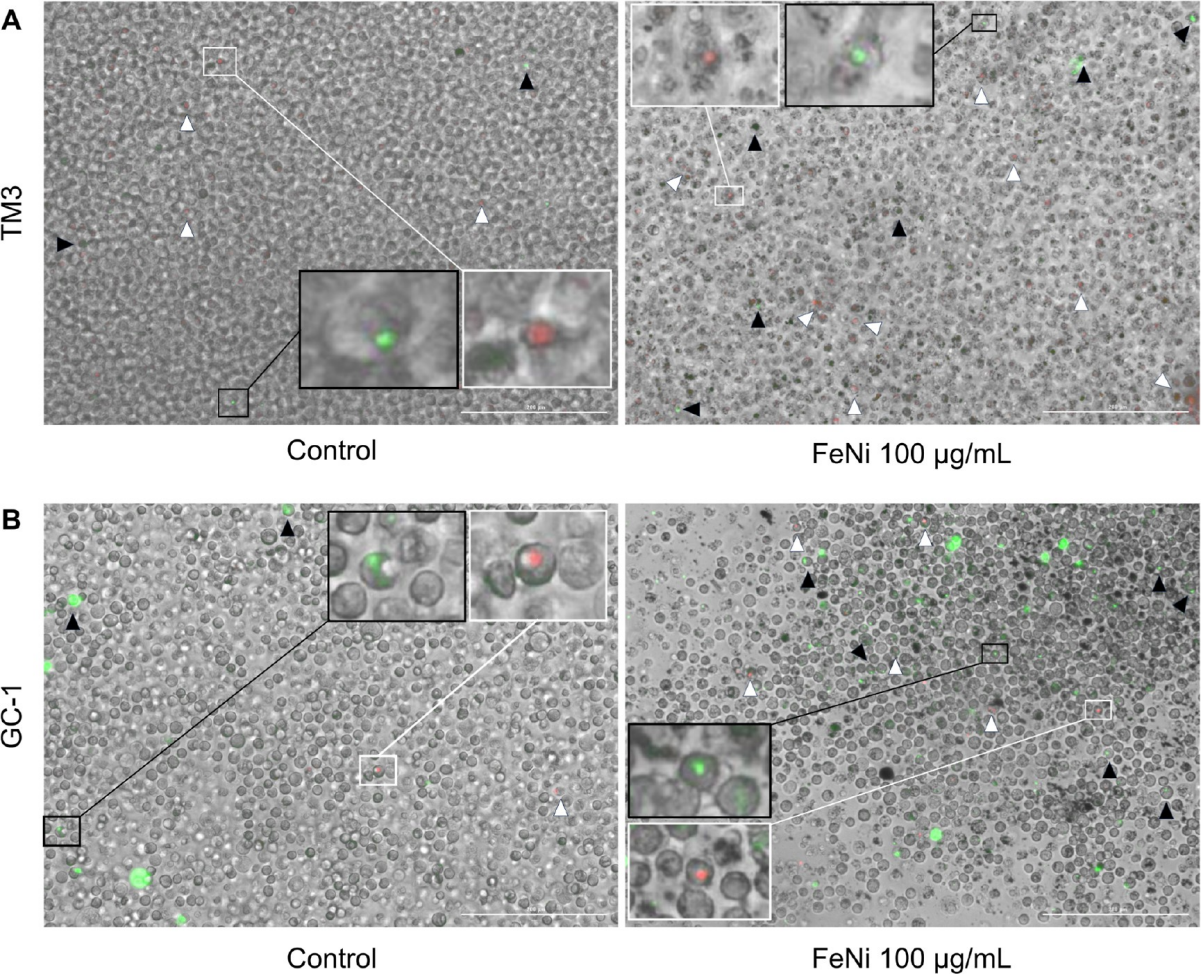
Annexin V/PI Staining. Staining of apoptotic and necrotic cells in TM3 cells (A) and GC-1 cells (B). Red fluorescence indicates the presence of propidium iodide (PI), evidenced by white arrowheads, demonstrating necrosis. Green fluorescence indicates the presence of Annexin V, evidenced by black arrowheads, identifying apoptotic cells. Enlarged sections allow better visualization of cells stained with Annexin V, highlighted in black boxes, while cells stained with PI are highlighted in white boxes. Images were acquired using the Cytation 5 Cell Imaging Reader (BioTek). Scale bar: 200 μm.

### 3.5 Intracellular Uptake of Nickel Ferrite Nanoparticles

#### 3.5.1 Magnetic Column

The kinetics of nanoparticle internalization into TM3 and GC-1 cells were assessed following exposures of 5, 10, 20, 40, and 60 min, as well as at 2 and 3 h. Notably, internalization was detectable as early as 5 min in both TM3 (Figure 5A) and GC-1 cells (Figure 5B). The efficiency of nanoparticle internalization was remarkable, reaching nearly 100% within just 2 h for both cell types (Figure 5A,B). Importantly, internalization remained at this maximum level with no significant changes observed at 24, 48, and 72 h of exposure to FeNi NPs (Supplementary Figure 2).

**Figure 5:**
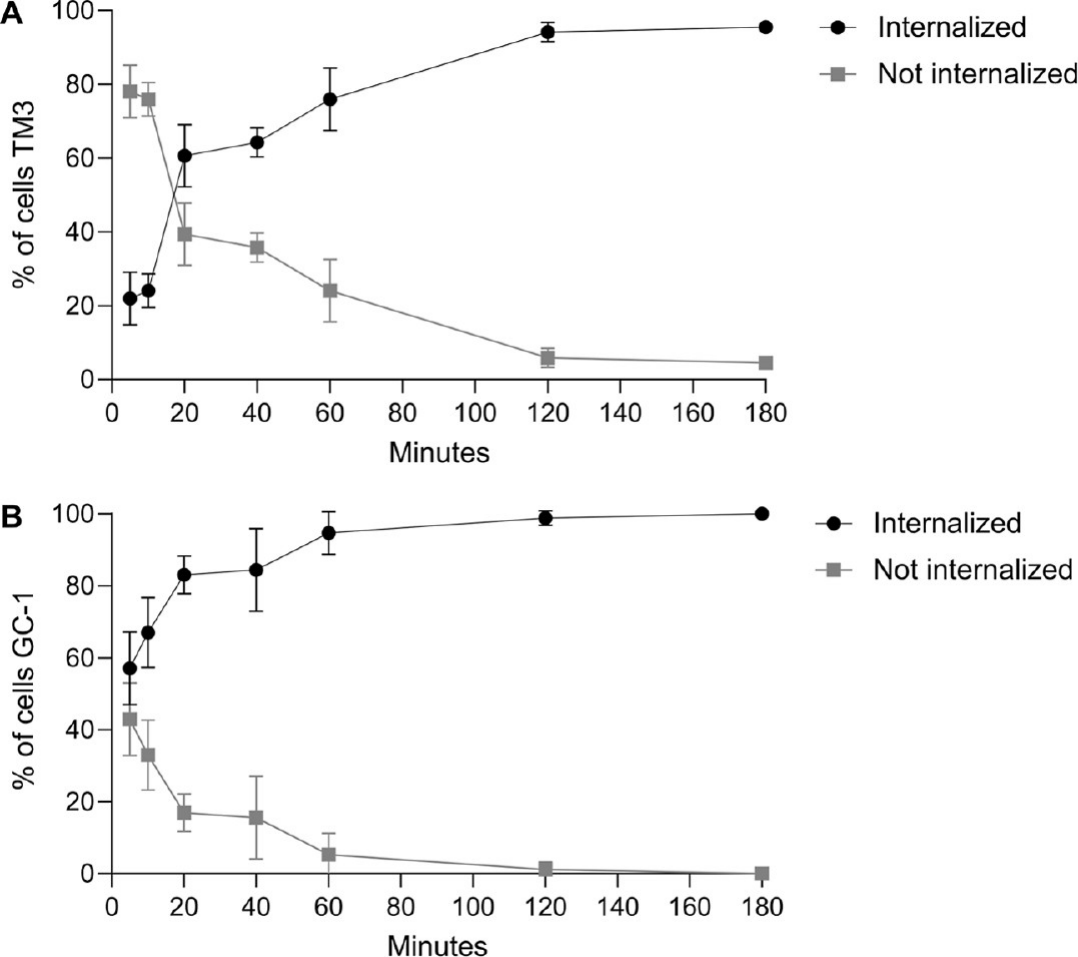
Internalization Kinetics. Internalization of nickel ferrite nanoparticles in TM3 cells (A) and GC-1 cells (B). The results were expressed as a percentage, represented by the ±SD (*n* = 3).

#### 3.5.2 TM3 Cells–Transmission Electron Microscopy

Transmission electron microscopy (TEM) analyses of TM3 cells revealed the presence of nickel ferrite nanoparticles in two distinct locations: (1) free within the cytoplasm, as indicated by the white circles (Figure 6A1); and (2) encapsulated within vesicles in the cytoplasmic region, indicated by the black arrows (Figure 6A2). These findings suggest that the internalization of FeNi nanoparticles by cells may occur via both passive diffusion and endocytosis. In addition to these mechanisms, the formation of a phagosome was observed in TM3 cells, containing FeNi NPs, as indicated by white arrows (Figure 6A3), suggesting they may also be phagocytosed. It is important to highlight the substantial presence of nanoparticles identified within the cytoplasmic environment, as illustrated in Figure 6A4. In this figure, FeNi nanoparticles are encircled in black, indicating a significant amount of nanoparticle internalization in TM3 cells.

**Figure 6:**
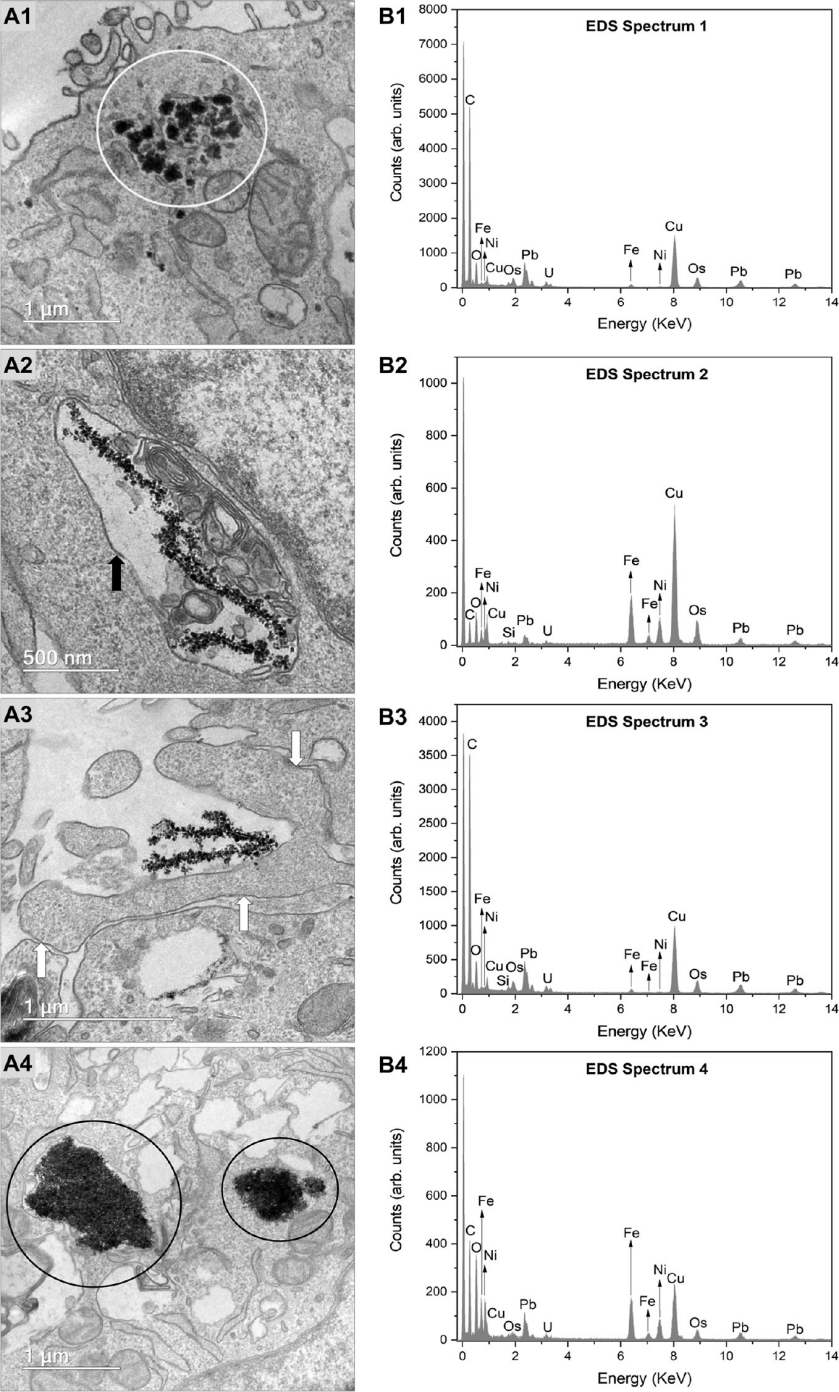
Intracellular internalization of nickel ferrite nanoparticles in TM3 cells. (A1–A4) Transmission electron microscopy (TEM) images demonstrating nanoparticle internalization in TM3 cells. (B1–B4) The composition of the nanoparticles was verified by energy-dispersive X-ray spectroscopy (EDS), correlated with the regions shown in the TEM images: A1 corresponds to B1, A2 to B2, A3 to B3, and A4 to B4. The presence of nanoparticles dispersed within the cytoplasmic region is indicated by white circles. Black arrows highlight vesicle membranes, representing the presence of intravesicular nanoparticles. Additionally, white arrows denote the formation of phagosomes encapsulating nickel–iron nanoparticles. Areas with a notable abundance of FeNi nanoparticles within the cytoplasm are indicated by black circles.

Energy-dispersive X-ray spectroscopy (EDS) played a pivotal role in confirming the identity of the nanomaterial, as it successfully detected the presence of iron (Fe) and nickel (Ni) across all analyzed fields for TM3 cells (Figure 6A1–A4). This analytical technique was essential to establish that the material evaluated corresponds to FeNi NPs. The spectra obtained for the TM3 cells correspond to the respective analysis fields: B1 corresponds to A1, B2 to A2, B3 to A3 and B4 to A4 (Figure 6A1–B4).

#### 3.5.3 GC1 Cells—Transmission Electron Microscopy

For GC-1 cells, a profile similar to that observed in TM3 cells was documented. FeNi NPs were identified as follows: (1) present freely within the cytoplasm, as indicated by white circles (Figure 7A5); and (2) enclosed within vesicles located in the cytoplasmic region, as demonstrated by black arrows (Figure 7A6,A7), which supports the hypothesis that passive diffusion and endocytosis may serve as potential pathways for cellular internalization. Figure 7A8 illustrates a significant abundance of FeNi NPs within the intracellular space of GC-1 cells, as highlighted by black circles, further confirming a high level of internalization. No FeNi nanoparticles were detected within the nuclei of either TM3 or GC-1 cells.

**Figure 7:**
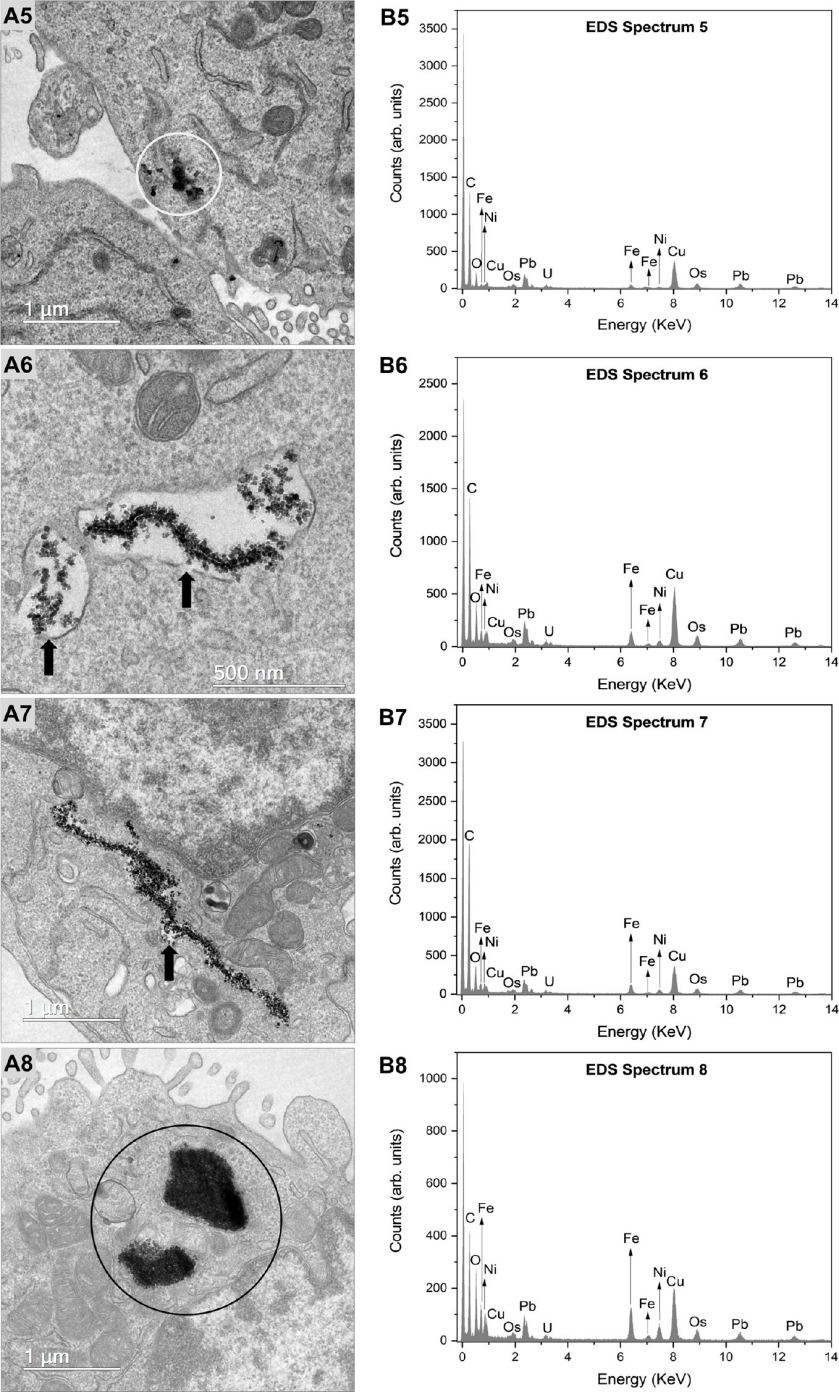
Intracellular internalization of nickel ferrite nanoparticles in GC-1 cells. (A5–A8) Transmission electron microscopy (TEM) images show the internalization of nickel ferrite nanoparticles in GC-1 cells. (B5–B8) The identity of the nanoparticles was confirmed by energy-dispersive X-ray spectroscopy, correlated with the regions shown in the TEM images: A5 corresponds to B5, A6 to B6, A7 to B7, and A8 to B8. The presence of nanoparticles dispersed within the cytoplasm is indicated by white circles. Black arrows highlight vesicle membranes, representing the presence of intravesicular nanoparticles. Areas with a notable abundance of FeNi nanoparticles within the cytoplasm are indicated by black circles.

Through energy-dispersive X-ray spectroscopy (EDS), the identity of the nanomaterial was further confirmed by the detection of Fe and Ni peaks in GC-1 cells, where B5 corresponds to A5, B6 to A6, B7 to A7, and B8 to A8 (Figure 7A5–B8). All spectra analyzed exhibited characteristic peaks of iron (Fe) and nickel (Ni), confirming the presence of the nanomaterial within the intracellular environment. Any additional elements detected were likely residues from sample preparation for TEM analysis or artifacts from the microscope itself.

### 3.6 FeNi NPs Increase ROS Production

After 72 h of exposure to nickel ferrite nanoparticles, ROS production was quantitatively assessed in TM3 and GC-1 cells using the DCFDA/H_2_DCFDA assay. Both cell lines exhibited significantly elevated ROS levels in the FeNi NP-treated groups compared to the negative control, with levels comparable to the positive control (H_2_O_2_-induced; Figure 8A–B). Qualitative analysis confirmed these findings, with green fluorescence signals (DCFDA) detected in both FeNi NP-treated and positive control groups, while minimal fluorescence was observed in the negative control (Figure 8C). Notably, the fluorescence intensity in FeNi NP-treated cells was more similar to the positive control than to the negative control in both cell types, indicating substantial ROS production induced by nanoparticle exposure.

**Figure 8:**
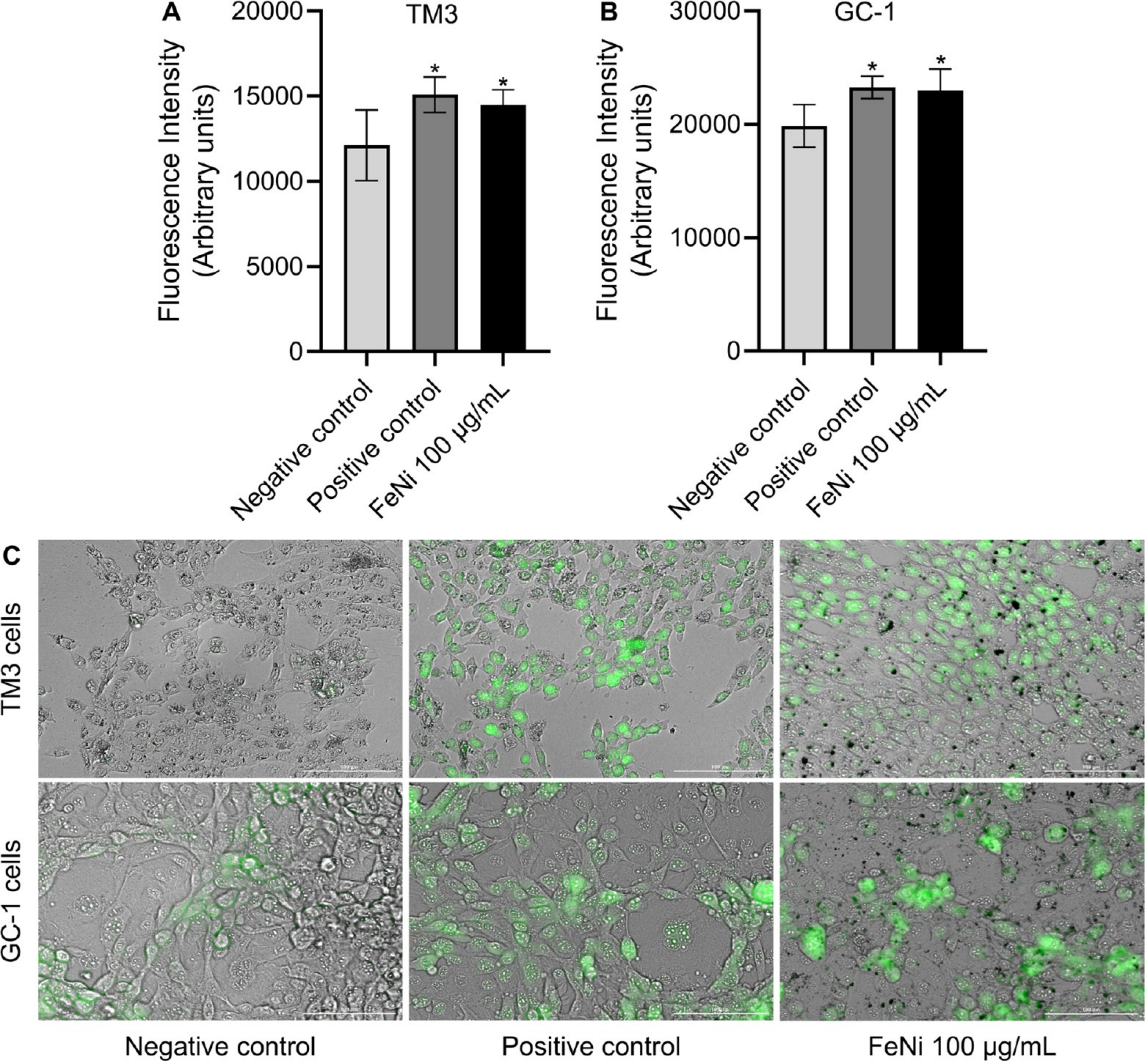
Reactive oxygen species (ROS) production in reproductive cells following 72 h of exposure to FeNi nanoparticles. (A,B) Quantitative ROS levels in TM3 and GC-1 cells, respectively, measured using the DCFDA/H_2_DCFDA assay kit. (C) Qualitative visualization of ROS production showing green fluorescence signals in TM3 and GC-1 cells exposed to FeNi nanoparticles (100 μg/mL), positive control (H_2_O_2_, 100 μM), and negative control. Scale bar: 100 μm. Data are expressed as mean ± SD (*n* = 6). Statistical analysis was performed using one-way ANOVA followed by Dunnett’s test. Differences were considered statistically significant at *p* ≤ 0.05 (*).

## 4 Discussion

The nickel ferrite NPs synthesized by the hydrothermal method presented high crystallinity, superparamagnetic behavior at room temperature, and low aggregation in the dispersions prepared for biological assays. At a dose of 500 μg/mL, FeNi NPs reduced the viability of all tested cell types, proving toxic to both systemic and male reproductive cells at all time points. Due to compromised viability in VERO cells at 250 μg/mL and in AML-12 cells after 72 h, we chose to use 100 μg/mL in our assays, as it is considered a safe dose for kidney and liver cells for upcoming in vivo experiments. We observed that the selected FeNi NP dose significantly increased cell death by apoptosis and necrosis in Leydig cells, and by apoptosis in germ cells. This pattern is interesting, as our experiments demonstrated greater resistance of GC-1 cells to FeNi NPs compared to TM3 cells. Ferraz (2020)^50^ observed the same pattern, showing higher sensitivity of Leydig cells to exposure to iron oxide NPs compared to germ cells. We observed rapid internalization of FeNi nanoparticles in both cell types within the first 5 min of exposure. Transmission electron microscopy confirmed their presence in the cytoplasm and within vesicles, suggesting internalization via passive diffusion and endocytosis. Phagosome formation was observed in TM3 cells. Furthermore, in TM3 and GC-1 cells, we observed an increase in ROS after exposure to FeNi NPs, demonstrating their adverse effects on male reproductive cells.

The nickel ferrite nanoparticles were characterized by X-ray diffraction, which confirmed their crystalline state and indicated a structural conformity to the typical cubic ferrite configuration. When dispersed in injection water, the zeta potential exceeding 30 mV, in conjunction with sodium coverage identified through X-ray photoelectron spectroscopy (XPS), suggested appreciable colloidal stability.^51^ In cell culture media, the zeta potential decreased, probably due to the formation of a protein corona, behavior commonly reported under physiological conditions.^38,39^ Nevertheless, dynamic light scattering (DLS) measurements demonstrating a hydrodynamic size below 50 nm indicated that the nanoparticles remained with a low degree of aggregation even in the culture medium, compared with the estimated nucleus size of 13.5 nm.^52^ The data also indicate that the nanoparticles exhibit superparamagnetic behavior without residual magnetism, thereby supporting their prospective applications in the biomedical field.^53^

The study findings indicated that nickel ferrite nanoparticles demonstrated toxicity that varied based on the dosage, exposure duration, and specific cell type. This observation aligns with the research conducted by Ferraz et al. (2020),^32^ who assessed the viability of human fibroblasts subjected to iron oxide nanoparticles using the CellTiter-Blue reagent. The results revealed that cell viability remained above 70% across the four evaluated cell lines (AML-12, ATCC CRL-2254, VERO, ATCC CCL-81, TM3, ATCC CRL-1714, GC-1, ATCC CRL-2053) when treated with a concentration of 100 μg/mL. According to ISO 10993-5 standards, a substance is classified as cytotoxic only if cell viability falls below 70%.^49,54^ Consequently, this concentration of 100 μg/mL has been selected for subsequent assays, as it presents lower risk to systemic cells in vitro. However, comprehensive in vivo studies will be essential to assess biodistribution, metabolism, and systemic side effects before any clinical application.

The biocompatibility of citrate-coated nickel ferrite nanoparticles^14^ and PEGylated nanoparticles^19^ has also been demonstrated at doses of up to 100 μg/mL in breast cancer cells, as observed in our assays. On the other hand, Ahamed et al., 2011 ^55^ demonstrated the cytotoxicity of nickel ferrite NPs in human pulmonary epithelial cells (A549) at concentrations between 25 and 100 μg/mL, showing a dose-dependent effect. This variation is expected, as ferrites can exhibit different effects depending on their physicochemical properties and the cell type used in the assay.^14^ Doses above 250 μg/mL demonstrated toxicity to both systemic and reproductive cells, with the dose of 500 μg/mL showing the most harmful effects across all cell types and time points analyzed. This confirms a dose-dependent effect, influenced by the concentration and exposure time to the sample.^3^ Research shows that superparamagnetic iron oxide nanoparticles reduce cell viability in primary cultures of Leydig, Sertoli, and male germ cells in a dose- and time-dependent manner, with the most significant toxicity observed in Sertoli and Leydig cells.^35^ In vivo studies also indicate these nanoparticles impair Leydig and germ cells, which reduces sperm production.^31^

Annexin V/PI staining revealed that FeNi nanoparticles induced apoptosis in both TM3 and GC-1 cells, with necrosis additionally observed in TM3 cells. Apoptosis is a form of programmed cell death.^56,57^ Ahamed et al. (2011)^55^ demonstrated that nickel ferrite nanoparticles induced the activity of apoptotic enzymes, caspase-3 and caspase-9, in a dose-dependent manner at doses ranging from 25 to 100 μg/mL in A549 cells. Other well-established nanoparticles, such as iron oxide nanoparticles, have also been shown to induce cell death, particularly apoptosis, in HT-29 cells^58^ and RAW264.7 macrophages in vitro,^59^ as well as to enhance apoptotic activity in the epididymis of male mice in vivo.^60^ Different types of NPs can induce cell death through either apoptosis or necrosis.^61^ GC-1 cells exhibited greater resistance with minimal necrosis, while TM3 cells showed increased necrotic cell death. Although necrosis induction by FeNi nanoparticles has not been extensively documented in vitro, it has been observed in rabbit tissues exposed to FeNi.^62^

After exposing cells to nickel ferrite nanoparticles, MACS technology was used to evaluate the internalization time of the nanomaterial, leveraging the nanoparticles magnetic properties demonstrated by VSM characterization and supported by previous studies.^14,18,19^ FeNi nanoparticles were observed within the cells as early as 5 min postexposure. Furthermore, following 2 h of contact, nearly all cells had internalized the nanomaterial, with internalization levels remaining stable at 24, 48, and 72 h, indicating sustained cellular retention. Ferraz et al. (2020)^32^ reported that iron oxide nanoparticles are internalized by fibroblasts within 15 to 30 min, influenced by their size, shape, and surface characteristics. The internalization of nanoparticles is currently a prominent area of research, particularly due to its significance in drug delivery applications.^63^ Furthermore, the concentration of nanoparticles within cells emerges as a critical factor; higher concentrations are associated with increased rates of internalization and may eventually reach a saturation point.^64^

TM3 and GC-1 cells displayed similar internalization patterns at 2 and 3 h of exposure. TEM analysis showed nickel ferrite nanoparticles in the cytoplasm, either dispersed or within vesicles, indicating two possible entry mechanisms: diffusion and endocytosis. Additionally, TM3 cells exhibited phagosome formation, suggesting FeNi nanoparticles may also undergo phagocytosis. Real-time imaging of labeled nanoparticles or pharmacological inhibitors targeting specific endocytic and phagocytic pathways would be valuable to precisely characterize these internalization pathways. Nanoparticle localization in cells can depend on size. In cancer cells, gold nanoparticles between 2 and 6 nm were found in both the cytoplasm and nucleus, while larger nanoparticles (15 nm) were only seen in the cytoplasm, often in aggregated form.^65^ Nanoparticles in the cytoplasm can interact with proteins and organelles, leading to increased cellular cytotoxicity.^66^ Ferraz et al. (2020)^32^ also reported the presence of iron oxide nanoparticles in endosomes and dispersed in the cytoplasm of human fibroblast cells.

As shown by Kückelhaus et al. (2003)^67^ and Ferraz et al. (2020),^32^ our citrate-coated cobalt ferrite and iron oxide nanoparticles were also not found in the cell nucleus. According to Oh and Park (2014),^63^ the surface charge of nanoparticles affects their endocytosis pattern. The observed surface sodium and positive zeta potential in our sample suggest that cellular internalization may have been enhanced. Positively charged nanoparticles enhance uptake due to their attraction to slightly negative cell membranes.^68^ Cell type and surface charge are crucial for nanoparticle internalization and biocompatibility, with surface charge playing a key role in cellular uptake.^69^

The high cellular internalization rate in Leydig and germ cells, along with the superparamagnetic properties of nickel ferrite nanoparticles, may support future in vivo studies on using magnetic hyperthermia to induce animal sterility. Ding et al. (2021)^70^ found that PEG-coated iron oxide nanoparticles reduced sperm count and motility after intratesticular injection, effectively controlling male animal conception. Similar research on citrate-functionalized manganese-ferrite nanoparticles has shown that exposure to a magnetic field can lead to seminiferous tubule degeneration, testicular atrophy, and reduced testosterone levels.^71,72^

Reactive oxygen species (ROS) are essential mediators of cellular signaling and homeostasis; however, excessive production leads to oxidative stress and cellular dysfunction.^73^ In this study, exposure to nickel ferrite nanoparticles significantly elevated ROS production in both TM3 and GC-1 cells compared to the negative control. This finding is consistent with previous reports demonstrating ROS-mediated oxidative stress induced by FeNi nanoparticles in various cell types. Ahamed et al. (2011)^55^ documented oxidative stress in human lung epithelial cells (A549) following FeNi NP exposure, while Vatan (2022)^74^ reported elevated intracellular ROS in cells treated with iron–nickel alloy nanoparticles. Similarly, nickel ferrite nanoparticles have been shown to induce oxidative stress in human hepatic (HepG2) and breast (MCF-7) cancer cells, as well as in HeLa cells exposed to nickel nanowires.^75,76^ Other nanoparticles, such as silica,^77^ iron oxide,^35,78^ and gold,^33^ similarly elevate ROS levels across diverse cell types. The mechanisms underlying nanoparticle-induced ROS generation are multifaceted, with distinct nanomaterials potentially activating different pathways.^73^

## 5 Conclusion

This comprehensive study successfully synthesized and characterized nickel ferrite nanoparticles with well-defined structural and magnetic properties. The nanoparticles exhibited a small cubic crystalline structure, superparamagnetic behavior at room temperature, and excellent colloidal stability in aqueous and cell culture media dispersions. Cytotoxicity assessment across reproductive and systemic cell types revealed dose- and time-dependent responses. VERO cells demonstrated the highest sensitivity, while reproductive cells (TM3, GC-1) and hepatocytes (AML-12) exhibited greater tolerance. Our data revealed that FeNi nanoparticles were rapidly and efficiently internalized, inducing apoptosis in reproductive cells. ROS generation emerged as a key mechanistic driver of cytotoxicity. These findings provide a foundation for future investigations into the biomedical applications of FeNi nanoparticles in reproductive cells.

## Supplementary Material



## Notes

Contains data regarding physicochemical behavior and biological responses to FeNi nanoparticles. Supplementary Figure 1 shows the colloidal stability profile of the nanoparticles in biologically relevant media, including hydrodynamic size and zeta potential measurements in different serum-supplemented culture media. Supplementary Table 1 contains the IC_50_ values obtained at 24, 48, and 72 h after nanoparticle exposure using all cell lines, with corresponding 95% confidence intervals, highlighting the differences in cytotoxic sensitivity among the cell lines evaluated. Finally, Supplementary Figure 2 includes the temporal kinetics of nanoparticle uptake, showing the percentage of TM3 and GC-1 cells that internalized the nanoparticles at 24, 48, and 72 h. Replicates were highly consistent, resulting in negligible standard deviation (PDF)
