# Supplementary material for: Biocompatibility of Nickel Ferrite Nanoparticles on Systemic and Testicular Cells
Source: ACS Omega. 2026 Jan 6;11(2):3067–83. doi: 10.1021/acsomega.5c09491 (PMC12824716; doi:10.1021/acsomega.5c09491)
Supplement: Supplementary file 1 [file ao5c09491_si_001.pdf]

Biocompatibility of Nickel Ferrite Nanoparticles on Systemic and Testicular Cells

Carla Cristina Martins Silva[1], Pedro Igor Macário Viana[1], Thalita Marcolan Valverde[1], José Domingos Ardisson[2], Daniele Alves Fagundes[2], Guilherme Mattos Jardim Costa[1]*

[1]Laboratório de Biologia Celular, Instituto de Ciências Biológicas, Universidade Federal de Minas Gerais - UFMG, Belo Horizonte - MG, Brasil.

[2]Laboratório de Síntese de Nanoestruturas, Centro de Desenvolvimento da Tecnologia Nuclear, Belo Horizonte - MG, Brasil.

*Corresponding author:

Guilherme Mattos Jardim Costa – gmjc@ufmg.br

Supplementary figures

A

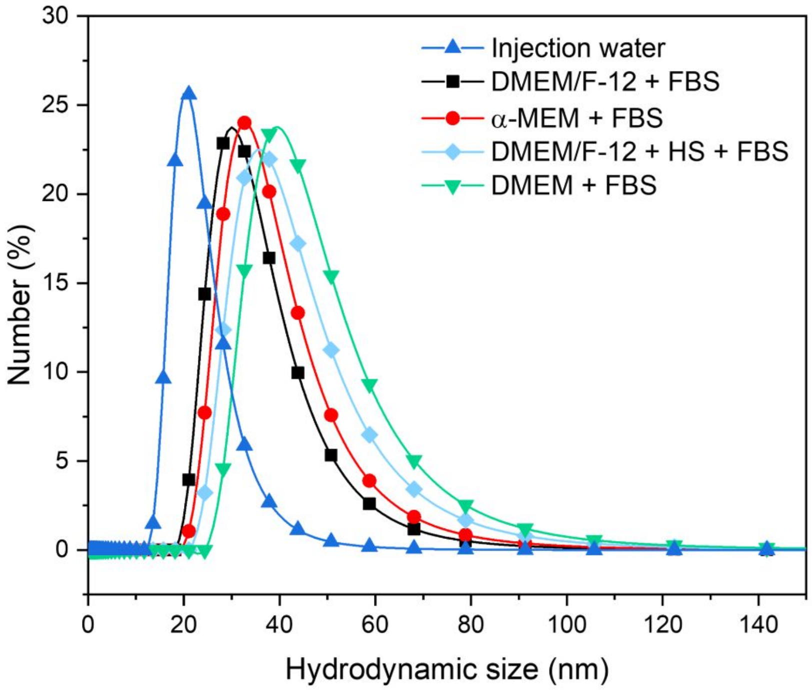

B

Sample	Hydrodynamic size (nm)	Zeta potential (mV)
Injection water	23.2 ± 0.5	38.0 ± 0.1
DMEM/F-12 + FBS	34.5 ± 6.0	-10.3 ± 1.1
α-MEM + FBS	38.2 ± 5.2	-11.2 ± 0.7
DMEM/F-12 + HS + FBS	43.9 ± 5.9	-7.8 ± 0.3
DMEM + FBS	42.0 ± 6.6	-11.3 ± 0.3

Supplementary Figure 1. Colloidal stability of FeNi nanoparticles in biologically relevant media. (A) Hydrodynamic particle size measured by dynamic light scattering (DLS) in injection-grade water and in cell culture media supplemented with fetal bovine serum (FBS): DMEM/F-12 + FBS (AML-12 cells), α-MEM + FBS (VERO cells), DMEM/F-12 + horse serum (HS) + FBS (TM3 cells), and DMEM + FBS (GC-1 cells). (B) Summary of hydrodynamic size (nm) and zeta potential (mV) measurements across all conditions. Measurements were performed in triplicate, and results are expressed as mean ± standard deviation.

Supplementary Table 1. Half-maximal inhibitory concentration (IC_{50}) values of nickel ferrite nanoparticles determined at 24, 48, and 72 hours of exposure in VERO, AML-12, TM3, and GC-1 cells. Data are expressed as mean IC_{50} values with corresponding 95% confidence intervals, reflecting the differential cytotoxic sensitivity among cell types and exposure timepoints.

	Hours	IC_{50} (µg/mL)	Confidence interval (95%)
VERO	24	283,4	224,3 to 358,2
	48	323,4	252,3 to 414,4
	72	297,4	209,9 to 421,3
AML-12	24	436,1	257,3 to 739,0
	48	549,0	275,6 to 1093
	78	536,3	295,6 to 973,0
TM3	24	493,8	381,8 to 638,6
	48	418,4	352,7 to 496,4
	72	529,5	384,0 to 730,2
GC-1	24	447,2	373,7 to 535,0
	48	433,3	376,2 to 499,1
	72	522,0	416,7 to 653,8

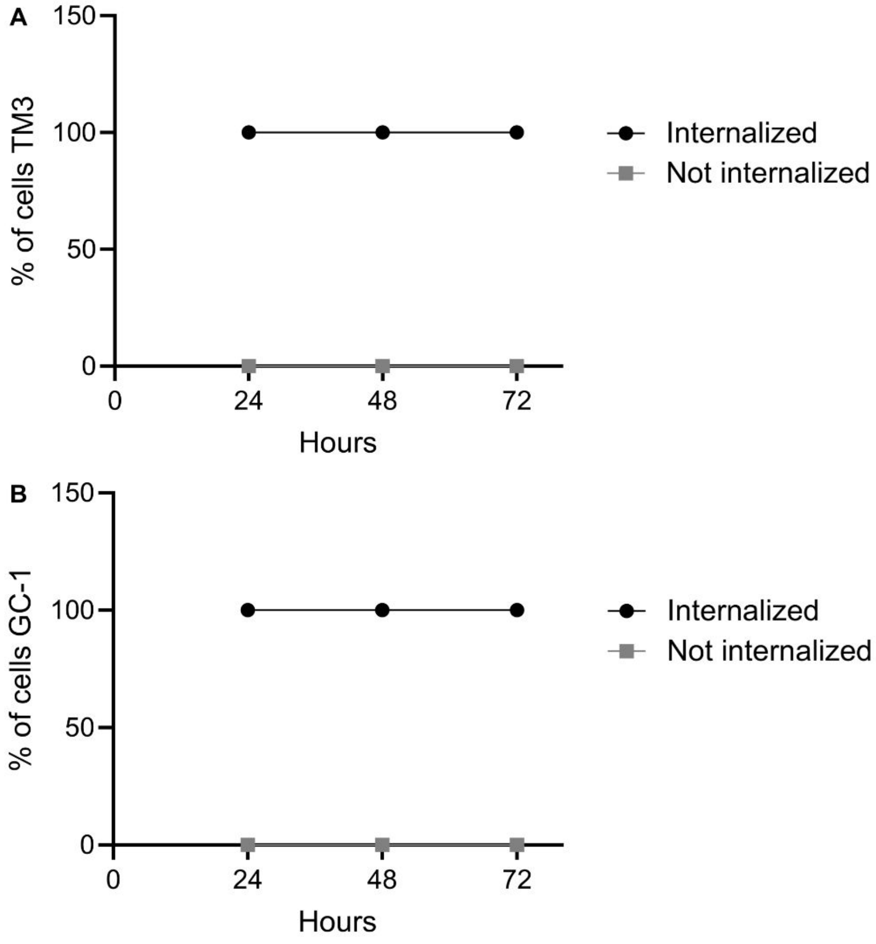

Supplementary Figure 2. Temporal kinetics of nanoparticle internalization. Percentage of cells internalizing nickel ferrite nanoparticles in TM3 (A) and GC-1 (B) cells at 24, 48, and 72 hours of exposure. Measurements were performed in triplicate (n = 3); standard deviation was negligible and is not visible in the figure due to high consistency across replicates.
